# Students' perception of a hybrid interprofessional education course in a clinical diabetes setting: a qualitative study

**DOI:** 10.5116/ijme.6165.59e0

**Published:** 2021-10-28

**Authors:** Mina Suematsu, Kenichi Okumura, Takeshi Hida, Noriyuki Takahashi, Kentaro Okazaki, Etsuko Fuchita, Keiko Abe, Hiroyuki Kamei, Manako Hanya

**Affiliations:** 1 Department of Education for Community-Oriented Medicine, Nagoya University Graduate School of Medicine, Nagoya, Japan; 2 Faculty and Graduate School of Pharmacy, Meijo University, Nagoya, Japan; 3 Ichinomiya Kenshin College, School of Nursing, Ichinomiya, Japan; 4 Department of Integrated Health Sciences, Nagoya University Graduate School of Medicine, Gerontological Nursing, Nagoya, Japan; 5 Clinical Nursing, Aichi Medical University College of Nursing, Nagakute, Japan

**Keywords:** Patient-centred care, interprofessional education, interprofessional work, asynchronous and synchronous online pro-grammes, hybrid learning, qualitative study

## Abstract

**Objectives:**

To explore what the student participants learned and how they felt about the use of three educational settings, namely, face-to-face workshop setting, asynchronous and synchronous online learning environments and interactions with outpatients in a real-world clinical setting in a hybrid interprofessional education course.

**Methods:**

This qualitative study used semi-structured in-depth interviews with healthcare undergraduate student participants in a course comprising workshops in three educational settings. A total of 15 healthcare undergraduate students, which included four medical, three pharmacy, five nursing and three nutrition students, completed this IPE course. All students agreed to participate in the study. We conducted four focus groups selected using convenient sampling. Focus group transcripts were analysed using the 'Steps for Coding and Theorization' qualitative data analysis method. We investigated the students' perception through the experience of three educational settings in the hybrid interprofessional education course.

**Results:**

The students recognised that this course had three types of educational spaces, namely, real, semi-real and unreal. Then, the positive changes in the awareness of students are trained in recognition of the patient perspective, the recognition of the roles discharged by the other professions and the recognition of the functions of their own profession after experiencing the educational spaces designated for this course.

**Conclusions:**

The repeated experience of participants to real, semi-real and unreal educational spaces promoted changes over time in the students' awareness of interprofessional competencies with respect to patient-centred care and ameliorated their readiness to undertake interprofessional tasks.

## Introduction

Interprofessional education (IPE) is essential for undergraduate students of healthcare professions for the worldwide improvement of the quality of patient care and to achieve patient-centred care.^1^^,^^2^ Multidisciplinary teams must expansively think in the context of medicine, as well as that of caregiving, and information must be freely shared between members and patients to accomplish interprofessional collaborative practices. However, multidisciplinary teamwork remains insufficient, according to the Commission on Education of Health Professionals for the 21st Century.^3^ Further, challenges requiring multidisciplinary medical teams have still not improved cooperation between team members primarily because of the inability of associates to apprehend their professional perspectives in a wider context.^4^ Numerous barriers to the implementation of IPE for undergraduate healthcare students persist despite the global requirement.^4^ Obstacles to IPE include the physical and temporal distances separating stakeholders charged with designing curricula in universities (barriers of time and space) and a mutual paucity of understanding resulting from cultural differences and chains of command (psychological barriers).^5^ During the COVID-19 pandemic, the need for online medical education programmes has been increasing worldwide and also has seen a growth in the use of asynchronous and synchronous information and communication technologies to deliver IPE. The online delivery of IPE can overcome scheduling and geographical challenges associated with interprofessional learning.^6^^,^^7^ Several recent studies have reported the efficacy of using Internet-based environments for learning.^8^ Although the online delivery of IPE recognises the alignment between the key principles of interprofessional learning and the collaborative constructivist approaches engaging groups of online learners in discourse and reflection,^9^ studies examining the effectiveness of IPE through Internet-based platforms remain scarce.^10^^-^^13^ Thus, we investigated student participants' perception of our hybrid IPE course in partnership with patients with diabetes that incorporated three types of educational settings: face-to-face workshop setting, asynchronous and synchronous online learning environments and interactions with outpatients in a real-world clinical setting based on student participants' narratives.

This study aimed to explore what the undergraduate healthcare professional students learned and how they felt about the use of the three educational settings.

## Method

### Study design, participants and setting

This study, which focuses on the experiences and perceptions of student participants in a hybrid interprofessional education course, used a qualitative design to explore what the student participants learned and how they felt about the use of three educational settings: face-to-face workshop setting, asynchronous and synchronous online learning environments and interactions with outpatients in a real-world clinical setting.

The study was conducted with healthcare professional undergraduate students who participated in the hybrid IPE course in partnership with patients with diabetes from October 17 to November 14. The 15 healthcare undergraduate students included four medical, three pharmacies, five nursing and three nutrition students. We selected all student participants by convenient sampling. An invitation to participate in this study was issued to all students who had just completed the hybrid IPE course.

Ethical approval was issued by the ethical committee of the Nagoya University School of Medicine, Japan, under its file number 2014-0220-2. All participants individually signed the informed consent forms regarding their agreement to participate according to the guidelines of brief descriptions to study subjects from the ethical committee of the Nagoya University School of Medicine, Japan.

### Details of the hybrid IPE course in partnership with patients with diabetes

The course was a hybrid IPE with three types of educational settings: face-to-face workshop setting, asynchronous and synchronous online learning environments and interactions with outpatients in a real-world clinical setting. The learning goal for the students was to offer an original educational programme during which students would deliver presentations as multidisciplinary teams for outpatients with diabetes in a real-world clinical setting. We used WebCampus as an asynchronous online module, and a free web conference system was downloaded as an asynchronous module.

It was offered as an optional course for one month; thus, motivated students who were interested in IPE volunteered for the programme. Initially, a one-day, in person workshop was held for all participating students on October 17. Presentations related to diabetes education were delivered by the students to outpatients with diabetes in a real-world clinical setting on November 14, the last day of the course. The students included 15 undergraduates enrolled in varied years of discrete programmes: four 5th-year medical students, three 5th-year pharmacy candidates, five 4th-year nursing apprentices and three 3rd-year nutrition scholars. The medical and nursing students came from Nagoya University, the pharmacy students were from Meijo University, and the nutrition students were from Nagoya University of Arts and Sciences. They were divided into three mixed-profession teams, and each team was required to discuss and create their original diabetes-related educational presentations.

Three teams delivered original educational programmes for outpatients with diabetes in the real-world clinical setting. Based on the previous experiences garnered in 2014, the teams focused on interactive approaches that were both easy to understand and enjoyable for participants.^14^ The first team created a quiz related to World Diabetes Day, intending to promote general awareness about diabetes management. The second team staged an original short skit with hypoglycaemia and how to handle this condition as its theme. The third team demonstrated sample exercises using both cognitive and physical aspects.

### Interviews and Data collection

Interviews were conducted with the participating students after they had experienced all the three educational settings of the IPE course. Convenient sampling was used to gather participants into four focus groups pertaining to the medical, nursing, pharmacy and nutrition disciplines and students were invited to participate in the study to represent their professions as focus group members. Students were accepted into their focus groups only after they had tendered their informed consent at the end of the IPE course. They were also apprised of their right to decline participation and withdraw at any juncture without needing to offer reasons for their refusal. The focus groups were executed by individual interviewers whose professions differed from the disciplines of the members of the respondent group: for example, a nursing educator interviewed the group of pharmacy students. This measure was taken to enable the interviewees to answer questions without fear or anxiety and avoid compromising the focus group because of a potential power imbalance between the interviewers and the respondent group.^15^ All the interviewers were also educators who were committed to the IPE course, and they used asynchronous and synchronous online modalities such as WebCampus and web conference system. They queried students about the conveniences and difficulties of those online modalities. The interviewers were trained using the same interview guides and performed the interviews according to a similarly established structure (Appendix). Interview guides were established through our repeated discussions of the effective methods of investigating the students' perception of the use of the three educational settings in this IPE course by the first, second, third and last authors. The one-time interviews took 30 minutes for each focus group, and all four interviews were simultaneously conducted in a separate area of the local hospital where the students delivered original educational programmes for outpatients with diabetes in a real-world clinical setting. All recordings were collected by the first author as m4a format data and shared with the second author for transcription.

### Data analysis

The recordings of focus group discussions were transcribed verbatim immediately after the interviews by the second author and checked by the first and last authors. The transcriptions were then qualitatively analysed using the 'Steps for Coding and Theorization (SCAT)', a four-step coding process used to identify themes and constructs, develop a storyline by weaving the themes and constructs and finally offer theories.^16^^,^^17^ SCAT was developed as an accessible qualitative data analysis method by T. Otani from Nagoya University in Japan.^16^^,^^17^ This method has been used by researchers in the fields of medical education and palliative care.^18^^-^^20^ The analysis consists of two main steps: decontextualisation to generate themes from sentences and then theorisation to construct theories summarising collected information. In the first decontextualisation process, we extracted keywords from original sentences, rephrased them using professional terms and created themes. In the second recontextualisation process, we developed a storyline from the emerging themes. Finally, we attained theorisation as a goal. This method was selected because of its explicit analytical process and its ability to accord researchers the opportunity to evince critiques and falsifiability, thus ensuring the validation of posited theories. The analysis was primarily conducted by the first, second and last authors and was checked by the other authors from the perceptions of each profession. When ambiguity was found in the analysis as part of the checking, the matter was discussed to achieve a consensus.

## Results

We recruited 15 participants for this study. All participants were simultaneously interviewed once after they had completed the IPE course. The median interview time was 35 minutes. Separate analyses were performed for each represented profession, and the obtained theoretical descriptions were classified based on similar content. As a result, the researchers were able to identify the students' recognition: the hybrid IPE course in partnership with patients with diabetes had three types of educational spaces, namely, real, semi-real and unreal, and the positive changes in the awareness of students after participating were attained in recognition of the patient perspective, the recognition of the roles discharged by the other professions and the recognition of the functions of their own profession after experiencing the educational spaces designated for this course. The detection of the above recognition was based on the narratives quoted below. A description of the concepts and text excerpts are given below (participant number, male/female, profession).

First, we identified the characteristics of the three educational spaces in the hybrid IPE course in partnership with patients with diabetes from the perspective of students. Each educational space was described below.

### Real educational space

The real educational space was made up of locations in which students could meet in person and perform group work. This space included face-to-face planned workshops and interactions with outpatients by delivering original educational programmes for outpatients with diabetes. Students were required to determine the schedules for sharing their workloads because it was mandatory for all members of a group to participate in the group tasks at the same time. Students were also required to establish the places where they would share workloads, gather and rehearse their group presentations, which would have to be performed in one set location. As the students talked face-to-face, they realised the merit of holding discussions while seeing each other's faces, and information was transmitted in real-time with no time lag. Consequently, students could bilaterally converse with each other and collaborate to cultivate three-dimensional discourses.

“I'm a pharmacy student. If the other students specialising in medicine, nursing and nutrition didn't get together or express their opinions from their disciplines, we wouldn't know until we met face-to-face”. (No. 4, male, pharmacy student)

“I think it was better that we all worked together in one place”. (No. 11, female, nursing student)

“It was a good experience, and it was not easy to work together with students from other faculties, such as the School of Medicine or the School of Nursing, unless we took part in something like this, so I would definitely recommend it”. (No. 5, female, pharmacy student)

“Well, I felt that there was a difference between doing it by correspondence and doing it directly”. (No. 6, male, pharmacy student)

“Surprisingly, we only met in person once, and we had web meetings only twice. Thus, we tend to be unprepared and anxious on the day of doing the presentation in front of the patients. We wish we had two or three more face-to-face meetings in spite of various constraints we were experiencing”. (No. 5, female, pharmacy student)

### Semi-real educational space

Semi-real educational space was so designated because they used web conference systems through which students could communicate via the use of monitors. In such space, the students were required to schedule sharing times and durations, but the web conference function allowed them to omit the establishment of a physical space where they would collaborate on their group tasks.

“Yes. It fits in with everyone's schedule”. (No. 1, female, nutrition student)

“On weekdays, when I came back from school, it was like 7 pm, so 8 pm was good for me”. (No. 3, female, nutrition student)

“I was totally fine with 10 pm, but there was one student who went to bed at 9 pm, so it was a bit of a burden for her”. (No. 2, female, nutrition student)

Communication occurred through monitors but remained face-to-face as in the real educational space, allowing bilateral conversations to proceed using gestures.

“All of us were displayed on monitors through cameras, and we could feel the presence of others more closely than when we communicated without monitors. I liked it”. (No. 11, female, nursing student)

This means of communication also allowed the real-time transmission of information. However, a system failure-related time lag or a delay in communication did occur during the use of the web conference system. This system failure resulted from the limited skills of faculty members with respect to the transmission of information and the different specifications of the personal computers used by the students.

“When I spoke, I was worried about the time lag, so I felt I could not speak well. I felt like I could not speak properly because of the time lag between when I spoke and when everyone else understood. When I tried to speak over other people while they were speaking, I felt like I was out of sync. My voice also sounded a bit late, which bothered me a lot and made it difficult to speak”. (No. 7, female, medical student)

“The time lag was huge. The specs of the computer belonging to each person and the speed of the lines were different, so the amount of data we could send also differed from person to person. Thus, this was messed up, and it was difficult to know who spoke first. It was a bit difficult to understand the order in which the conversations were flowing”. (No. 10, male, medical student)

Although the faculty members previously used an instruction manual to educate themselves with respect to the web conference system and shared this knowledge among themselves, the system did not work as intended. Hence, further improvement was indicated.

The semi-real educational space was thought to offer greater facilities in comparison to the real one. However, when students temporarily lost face-to-face communication once they began using the web conference system, they felt as if they had never met before despite previously meeting in person at the planned workshop.

“In the beginning, maybe because it was the first group work, we all were nervous and quiet”. (No. 1, female, nutrition student)

“It was the first experience for us to join in a web conference. As no one knew the procedure well, we could not log in. When we first met and talked at the web conference, we felt like, oh, we did not know each other. Since we had never met using web conference, we were anxious”. (No. 2, female, nutrition student)

However, as the web conference progressed, face-to-face discussions advanced and bilateral conversations were promoted, leading to the resumption of interpersonal communication as if the students were continuing face-to-face discourses with people they knew.

“Active statements were helpful in terms of time and progress”. (No. 1, female, nutrition student)

### Unreal educational space

The so-called unreal educational spaces allowed participants to chat, post comments and share files on WebCampus. Such spaces did not need the determination of times or locations for information sharing to occur, and students could access or upload information at all times.

“At each point, everyone presented their own versions, as needed. Someone presented an up-dated or revised version, and then others could present theirs one after another”. (No. 7, female, medical student)

On the other hand, real-time information transmission was not possible in the unreal educational spaces. The information to be transmitted accumulated until the receiver noticed the aggregation of unread comments.

“Even when someone had left a comment, I couldn't read it until I logged in”. (No. 8, female, medical student)

Thus, person-dependent time lags could occur in information sharing. In addition, conversations tended to unilaterally develop because students could not communicate face-to-face with each other.

“No one responded to the professor's comment until the last minute when it was noticed”. (No. 8, female, medical student)

“Sometimes, there was a complicated situation when using social networking services through a mobile phone”. (No. 1, female, nutrition student)

“I think we had improved learning when we gathered and worked together in one place”. (No. 11, female, nursing student)

Second, we identified the positive changes in the awareness of students after experiencing the educational spaces designated for the diabetes IPE course. After experiencing each educational space, the following types of awareness modifications were observed in the students who participated in the diabetes IPE course: the recognition of the patient perspective, the recognition of the roles discharged by the other professions and the recognition of the functions of their own profession.

### The recognition of the patient perspective

In the beginning, when students first attended the planned workshop in the real educational space, they noted the need to use exaggerated gestures to adjust the volume of their vocal communication, improve brochure designs or adopt other measures to address age-related physical dysfunctions when treating patients.

“We held a short skit with hypoglycaemia. When we rehearsed, we practised speaking slowly using exaggerated body and hand gestures for older patients”. (No. 14, female, nursing student)

However, the first planned workshop was held without patients, and the participating students were unable to accord careful consideration to the perspective of patients, such as making documents more legible. Similarly, the students could not acquire positive awareness on patient perspectives during the web conference within the semi-real educational space or through WebCampus chats in the virtual educational space, both of which occurred either without patients or without careful consideration of their needs.

“We realised that we did not visualise the distribution, as expected. I considered that our preparation might have been insufficient”. (No. 14, female, nursing student)

On the other hand, when the students ultimately delivered an original educational programme for people with diabetes in a real educational space, the students could sympathise with patients and establish close relationships with them because of their experience of the semi-real and unreal educational spaces.

“It was nice to see the patients having fun”. (No. 15, male, nursing student)

The students also became aware of the inadequacies of their explanations to patients, of which they were previously unaware as they prepared for their diabetes educational pro-grammes and began to reflect on their preparatory approaches.

“I noticed that some patients may also have difficulty in distinguishing colours. I should pay more attention to such patient traits”. (No. 13, female, nursing student)

Recognition of the roles discharged by the other professions

Before their participation in the planned workshop in the real educational space, students had limited opportunities to engage in daily exchanges with peers in other professional courses. They also felt the insufficient experience of interprofessional collaboration. Thus, the students acknowledged their insufficient understanding and recognition of the functions of the other professions.

“I think it was good to have the opportunity to communicate with students belonging to other professions. Besides, we rarely have opportunities to collaborate with each other to perform one activity”. (No. 9, male, medical student)

In contrast, although they communicated through monitors in the semi-real educational space, the students could engage in face-to-face interpersonal conversations, both in groups and bilaterally, which augmented their interest in their peers studying other professional courses and allowed them to compare their knowledge spans. Such appraisals enhanced their sense of fellowship and motived them further to prepare for the diabetes educational programme they would conduct as a team.

“I thought I should study harder to reach their level. As those of my profession do not study medicine so much in detail, their extensive knowledge impressed and stimulated me to study harder”. (Nos. 1, 2 and 3, female, nutrition students)

Although every conversation in the virtual educational space was unilateral, the file-sharing system helped the students understand the viewpoints of other disciplines. Consequently, psychological barriers to IPE were reduced in each participant.

'The whole experience gave me a good opportunity to understand the perspectives of students from other professions”. (No. 8, female, medical student)

When they ultimately delivered their diabetes educational programme as a group in the real educational space, the students realised the importance of collaborative work and the practices of other specialities.

“Working and creating together, I also became interested in, and learned about, their speciality areas”. (No. 12, female, nursing student)

“There are three types of care for diabetes: exercise, medication and diet. The three educational programmes were performed by multidisciplinary teams. So, holding an educational programme for patients with diabetes through collaborations among them might have been useful to effectively convey the details of such care to patients”. (No. 12, female, nursing student)

No negative recognition of peers of other disciplines was noted in the students regarding the three educational spaces that were used to accomplish the diabetes educational programmes.

### Recognition of the functions of their own profession

The planned workshop in the real educational space made the students realise the difficulties of explaining their own disciplines to peers belonging to other professions. They confronted problems in collaborating with their peers in other disciplines and attaining mutual understanding.

“Indeed, we had a hard time trying to provide students belonging to other professions and patients with an understandable (but not too abstract) explanation of something we identify as a matter of course; however, they did not know at all”. (No. 15, female, nursing student)

In the semi-real and unreal educational spaces, the students admitted to differences in opinions among the different disciplines vis-à-vis consideration for patients and discovered the need to explore appropriate methods of sharing information.

“How to communicate with patients, I thought that this was the focus of all professionals when adopting approaches. However, I also learned the differences among them will become visible with time and that I should be aware of this”. (No. 11, female, nursing student)

When the students ultimately presented their group diabetes education programme in the real educational space, they experienced a shift in roles from learners to teachers and encountered real-time reactions from patients. This experience granted them the recognition of the usefulness of their discipline for education of patients with diabetes and developed a sense of accomplishment within them.

“One of the merits of participating in this IPE course was the opportunity to relearn by teaching others about diabetes”. (No. 9, male, medical student)

Participating in delivering original educational programmes for outpatients with diabetes helped students redefine their own disciplines. Thus, they gained a sense of pride, and their awareness of the functions performed by professionals was enhanced. They became aware of the need to improve their skills.

“The education session for patients with diabetes made me realise the importance of our speciality area and increased my motivation to become a professional specialising in it. That stimulated me to study harder”. (No. 2, female, nutrition student)

Through their experience of the whole IPE course, the students evinced no negative recognition of their peers specialising in other disciplines.

## Discussion

Varied barriers have been reported in the execution of IPE; however, only a few studies have elucidated the enablers of such endeavours.^4^^,^^5^ The present study investigated the perception of the student participants in the hybrid IPE course in partnership with patients with diabetes using asynchronous and synchronous online modalities. Our results showed that this hybrid IPE course encompassed three educational spaces, namely, real, semi-real and unreal and illuminated the characteristics of each space. Each of these spaces evinced some obstructive and enabling features to execute interprofessional collaborative practice. Based on the outcomes of the applied course, this section discusses the challenges and applicability of IPE that uses all the three abovementioned educational spaces.

Temporal and physical barriers were noted when the IPE course on diabetes was first offered in 2014.^14^ Asynchronous, and synchronous online modalities were then adopted to remove these obstacles. The use of virtual educational spaces, such as the chatting function of WebCampus via Internet access, resolved the temporal and physical barriers. The use of the online platform also diminished psychological barriers among students specialising in entering discrete professions. Semi-real educational spaces, such as the web conference system, required students to set specific schedules for their discussions; however, virtual face-to-face discussions facilitated their preparations for their group education sessions for a patient with diabetes. The use of WebCampus chat and upload functions took away the need for students to set aside a time and place for the sharing of information, increased their opportunities for interpersonal communication, removed varied barriers between them and thus facilitated IPE. Previously conducted studies have also evidenced that online IPE mitigated temporal, physical and psychological barriers.^6^^,^^7^^,^^21^ The results obtained by this study were in line with those of previous studies and supported the efficacy of using semi-real and virtual educational spaces as auxiliary virtual locations where students enrolled in universities separated by temporal and physical distances could engage in IPE.

Nonetheless, the current study also identified supplemental difficulties, such as the tele-communication system failures that occurred during web conference sessions. A survey on the information technology literacy of undergraduate students revealed high Internet usage rates in students; however, the proportion of learners able to appropriately install software, connect/set up peripheral devices based on instruction manuals and manipulate new software using help functions or instruction manuals was found to be quite low, even in the developed countries.^22^ Students generally find devices such as smartphones that they frequently use in daily life easy to manipulate; however, many learners tend to resist unfamiliar equipment or gadgets they less frequently use. Therefore, educators must contemplate appropriate methods of employing personal computing environments and ascertain their students' information literacy when using social networks for their curricula. It may also be necessary for educators to assist student learning about information media for such purposes, especially in overcoming the problems posed by the current and urgent COVID-19 crisis.^23^

Real or semi-real educational spaces allow face-to-face discussions and are thus more desirable to achieve mutual understanding in students. Nonetheless, unreal educational spaces were also found to be effective in reducing psychological barriers because they helped students establish favourable relationships with peers in other disciplines and encouraged learners to modify their awareness of their own specialisations. In other countries, a group of pharmacy and nursing students of different universities created care plans for patients with diabetes using a web conference system and became cognisant of the roles discharged by the other speciality.^24^ In this IPE course discussed in the present paper, the update notification function allowed bilateral, rather than unilateral, chats, file-sharing and conversations in the unreal educational space, eliminating the need to schedule information sharing times and places. Dynamic interactions among students with different specialities may also be achieved using such methodology; however, this course was designed only to grant students the opportunity to apprehend the viewpoints of their peers in other disciplines and reduce psychological barriers between learners of discrete academic domains. The actual experience of interactions with patients with diabetes as a part of real educational spaces helped students sympathise with patients and transformed their understanding of their own specialities along with their perception of other disciplines. Thus, repeated access to the three educational spaces caused successive modifications in student awareness. Further, the component of educating patients as a part of the course granted students the opportunity to gain an understanding of the approaches adopted by other specialists in communicating with patients. In delivering their presentations, the students were also directly able to observe the reactions of patients, such as pleasure and confusion, which further encouraged them to improve their approach. Consequently, students could establish close relationships with patients and could appreciate the significance of their academic disciplines. A sense of accomplishment was thus inculcated in the participating students through the group delivering diabetes educational presentation for people with diabetes. Those experiences in the real educational space accorded students the opportunity to gauge the pleasure of patients in real-time and may have stimulated interactions among them that transcended their individual academic disciplines, thus guiding them towards a new phase of conceptual knowledge.^25^^,^^26^

IPE acquired through face-to-face communication in the real educational space tends to promote dynamic interactions both between the participating learners of varied disciplines and between patients and student participants.^27^^,^^28^ However, such opportunities cannot be frequently offered to students and patients. Semi-real and virtual educational spaces should, therefore, regularly be incorporated into courses to promote such beneficial interactions. The Japanese Ministry of Health, Labour and Welfare has highlighted the significance of medical information sharing networks and encouraged the IT-based prevention of more severe conditions through liaison systems for community-based integrated care.^29^ Social networks are also expected to contribute to advances in home care because the number of people unable to visit outpatient clinics will increase with the rapid ageing of the population in the future.^30^ Healthcare professionals will need to regularly communicate via social networks with other types of specialists and patients in such medical environments. Undergraduate education must soon address this additional prospective challenge.

### Limitation

Before concluding, a few shortcomings of the present study must be acknowledged. First, the optional curricular characteristic represents the principal limitation of this study. Only students interested in IPE would have volunteered for the course. Thus, the appreciation registered by the participants for this IPE course may be influenced by their internal motivations. Second, participants were required to recall previously acquired knowledge because the interviews were conducted after the IPE course was completed. Therefore, the feelings they expressed during the interviews could have differed to some extent from their emotions immediately after they experienced each of the three educational spaces. In spite of these drawbacks, the findings of this study will be useful for the amendment of the current IPE programme to suit the online educational mandates imposed by the COVID-19 pandemic.^31^

## Conclusions

The use of asynchronous and synchronous online modalities for the hybrid IPE course in partnership with patients with diabetes enhanced communication among students who attended different universities and facilitated interprofessional collaborative learning and practice by diminishing temporal, spatial and psychological barriers between participants. The repeated access of participants to real, semi-real and unreal educational spaces promoted changes over time in the students' awareness of interprofessional competencies with respect to patient-centred care and ameliorated their readiness to undertake interprofessional tasks. Other curricula are also expected to adopt collaborative online learning systems, such blended online modalities, in the future. To counteract the global burden of the COVID-19 pandemic, such courses should be developed taking into account the social networking environments and information literacy of the participating students.

### Acknowledgements

We thank all students who participated in the interviews and all faculty members who facilitated this IPE course. We extend our gratitude to the patients with diabetes who participated in the real-world clinical setting for this study and offer their appreciation to all the clinicians who undertook the physical care of the patients during this study at the local hospital.

### Conflicts of Interest

The authors declare that they have no conflict of interest.

## Supplementary material

Supplementary file 1 Appendix. Interview guide for four focus groups
