# Supplementary material for: Students' perception of a hybrid interprofessional education course in a clinical diabetes setting: a qualitative study
Source: Int J Med Educ. 2021 Oct 28;12:195–204. doi: 10.5116/ijme.6165.59e0 (PMC8995017; doi:10.5116/ijme.6165.59e0)
Supplement: Supplementary file 1 — Appendix. Interview guide for four focus groups [file ijme-12-195-S1.pdf]

Appendix

Interview guide for four focus groups

- How did you feel when participating in the hybrid IPE course in partnership with patients with diabetes?
  - What were the good things for you?
  - What were your concerns?
  - Why did you feel that way?
- How did you feel about actually doing the diabetes education programme yourselves when performing it?
  - What worked well for you?
  - What went wrong for you?
  - Why did you feel that way?
- How did you find the use of online modalities?
  - What were the good things for you?
  - What were your concerns?
  - Why did you feel that way?
- Would you recommend the diabetes IPE course to younger students?
  - Why did you feel that way?
- How did you find the communication with students from other professions?
